# Case report: The presence of third-degree atrioventricular block caused by pulmonary embolism masquerading as acute ST-segment elevation myocardial infarction

**DOI:** 10.3389/fcvm.2023.1013727

**Published:** 2023-08-08

**Authors:** Min Ma, Shichu Liang, Yong He, Hua Wang

**Affiliations:** ^1^ Department of Cardiology, West China Hospital, Sichuan University, Chengdu, China; ^2^ Department of Cardiology, The Sixth People’s Hospital of Chengdu, Chengdu, China

**Keywords:** acute myocardial infarction, third-degree atrioventricular block, pulmonary embolism, imaging, intracerebral hemorrhage

## Abstract

**Background:**

Pulmonary embolism (PE) typically presents with chest pain, tachypnea, hemoptysis, syncope, and increased markers of myocardial injury. On an electrocardiogram (ECG), sinus tachycardia, right bundle branch block (RBBB), S1Q3T3 pattern, and/or precordial T-wave inversion may be observed. Despite being one of the common causes of chest pain, a third-degree atrioventricular block (III° AVB) is rare in cases of PE, which can lead to difficulties in diagnosis or even overlooking this condition.

**Case summary:**

In this case report, we present a patient who was transferred to our hospital with suspected acute myocardial infarction (AMI). The patient's ECG showed ST-segment elevation in the inferior wall and a III° AVB, combined with significantly increased markers of myocardial injury. Interestingly, the patient also had a history of cerebral hemorrhage (ICH) for 7 days prior to being transferred to our hospital. After undergoing a systematic examination and evaluation, the final diagnosis for the patient was PE.

**Conclusions:**

In addition to considering common symptoms, it is important not to overlook rare symptoms when diagnosing a disease. This case serves as an example of how the misdiagnosis rate for PE can be reduced by conducting a comprehensive clinical evaluation and paying attention to all clinical clues and examination results.

## Introduction

Most cases of pulmonary embolism (PE) present with typical clinical manifestations such as chest pain, tachypnea, hemoptysis, and occasionally syncope. Additionally, on the electrocardiogram (ECG), characteristic changes can be seen in PE, including sinus tachycardia, right bundle branch block (RBBB), S1Q3T3 pattern, and/or precordial T-wave inversion (1). Abnormal Q waves may also appear on leads III and aVF on the ECG. However, in the case we report, none of these commonly seen features were observed. Instead, the patient presented with inferior wall ST-segment elevation, significantly increased myocardial injury markers, and a unique finding of third-degree atrioventricular block (Ⅲ° AVB). Initially suspected to be experiencing acute myocardial infarction (AMI), the patient underwent a thorough examination, which eventually led to the diagnosis of PE.

## Case presentation

**Figure 1:**
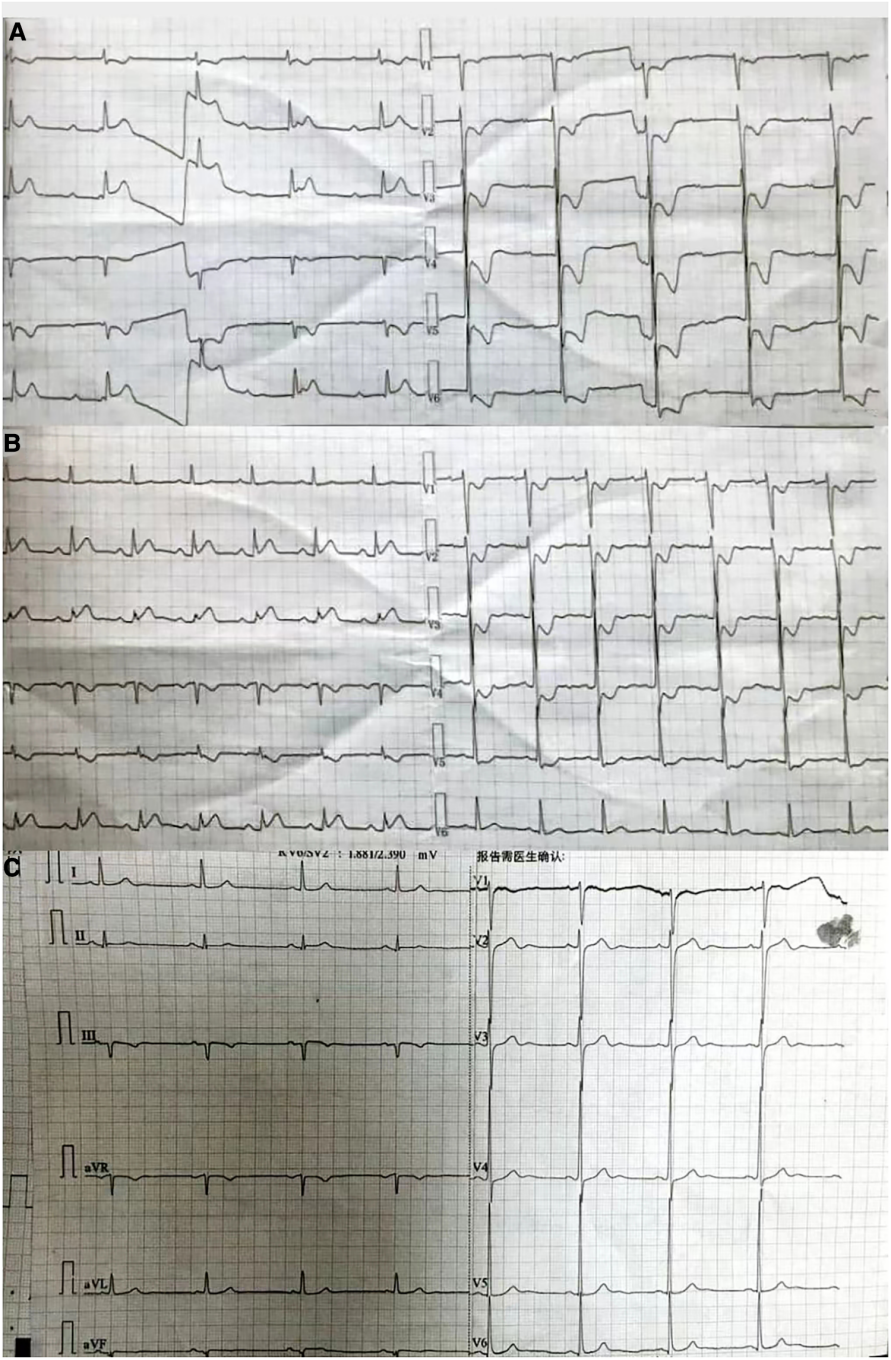
(**A**) ECG obtained at the local hospital. The findings suggest that the patient has been diagnosed with third-degree atrioventricular block. In addition, there are significant ST segment elevations observed in leads II, III, and aVF, and inverted T waves seen in leads V1–V6, accompanied by ST-segment depression. (**B**) ECG obtained at the emergency department of our hospital. The findings suggest that the patient has a sinus rhythm. There are inverted T waves observed in leads V1–V4, accompanied by a lesser degree of ST-segment depression. (**C**) ECG obtained at the Department of Cardiology of our hospital. The findings indicate left ventricular high voltage. There is a resolution observed in the ST segments of leads II, III, and aVF.

**Figure 2:**
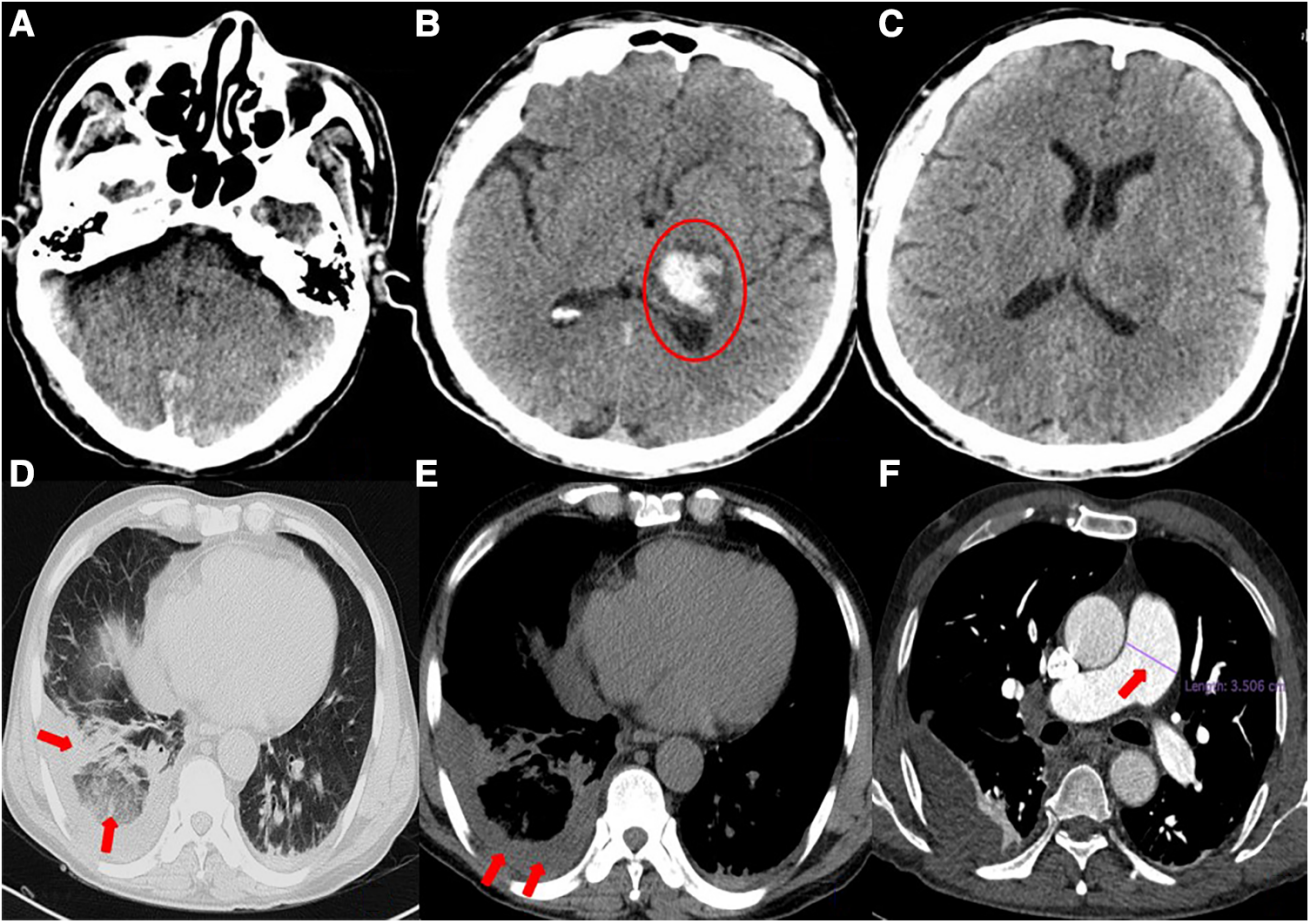
(**A–C**) General cranial computed tomography scan. A 3.3 × 2.3-cm left thalamic hematoma (red circle) is visible, with minimal surrounding edema. There is no evidence of midline shift. (**D–F**) Chest computed tomography scan. Scattered infection foci in bilateral lungs (especially in the lower lobes of both lungs), thickened bilateral pleura, right lower lobe atelectasis, right pleural effusion, and suspected pulmonary infarction can be seen. The pulmonary artery trunk is enlarged (red arrow).

A 54-year-old man was transferred from the local hospital to our hospital's chest pain center in the emergency department due to suspected AMI. The ECG obtained at the local hospital showed ST-segment elevation in the inferior wall leads (II, III, and aVF), indicating possible damage to the heart muscle in that area and a complete atrioventricular block (Figure 1A). When the patient arrived at our emergency department, another ECG was performed, which showed a sinus rhythm with ST-segment elevation in the inferior wall leads (Figure 1B). The patient did not exhibit obvious difficulty in breathing. The laboratory results showed a high percentage of neutrophils (84.3%), indicating an inflammatory response in the body. Furthermore, the results of biomarker analysis were as follows: cardiac troponin-T (cTnT) level, 474.4 ng/L; myoglobin (Mb) level, 565.30 ng/ml; creatine kinase MB (CK-MB) level, 108.00 ng/ml; and brain natriuretic peptide level, 264 pg/ml. Moreover, the patient underwent general cranial computed tomography (CT) scans (Figures 2A–C) and chest CT scans (Figures 2D–F) to assess any further complications or conditions.

Six days prior to admission to our hospital, the patient was admitted to the local hospital with symptoms of headache, right limb immobility, unclear speech, nausea, and vomiting. His blood pressure was measured at 180/120 mmHg and a cranial CT scan revealed intracerebral hemorrhage (ICH) in the thalamus. As a result, the patient was diagnosed with ICH. During his hospital stay, he experienced precordial squeezing chest pain, which lasted for approximately 1.5 h. This chest pain was accompanied by diaphoresis, palpitations, dyspnea, nausea, and vomiting. Given the symptoms and ECG changes indicating inferior wall involvement, acute inferior myocardial infarction were suspected.

**Figure 3:**
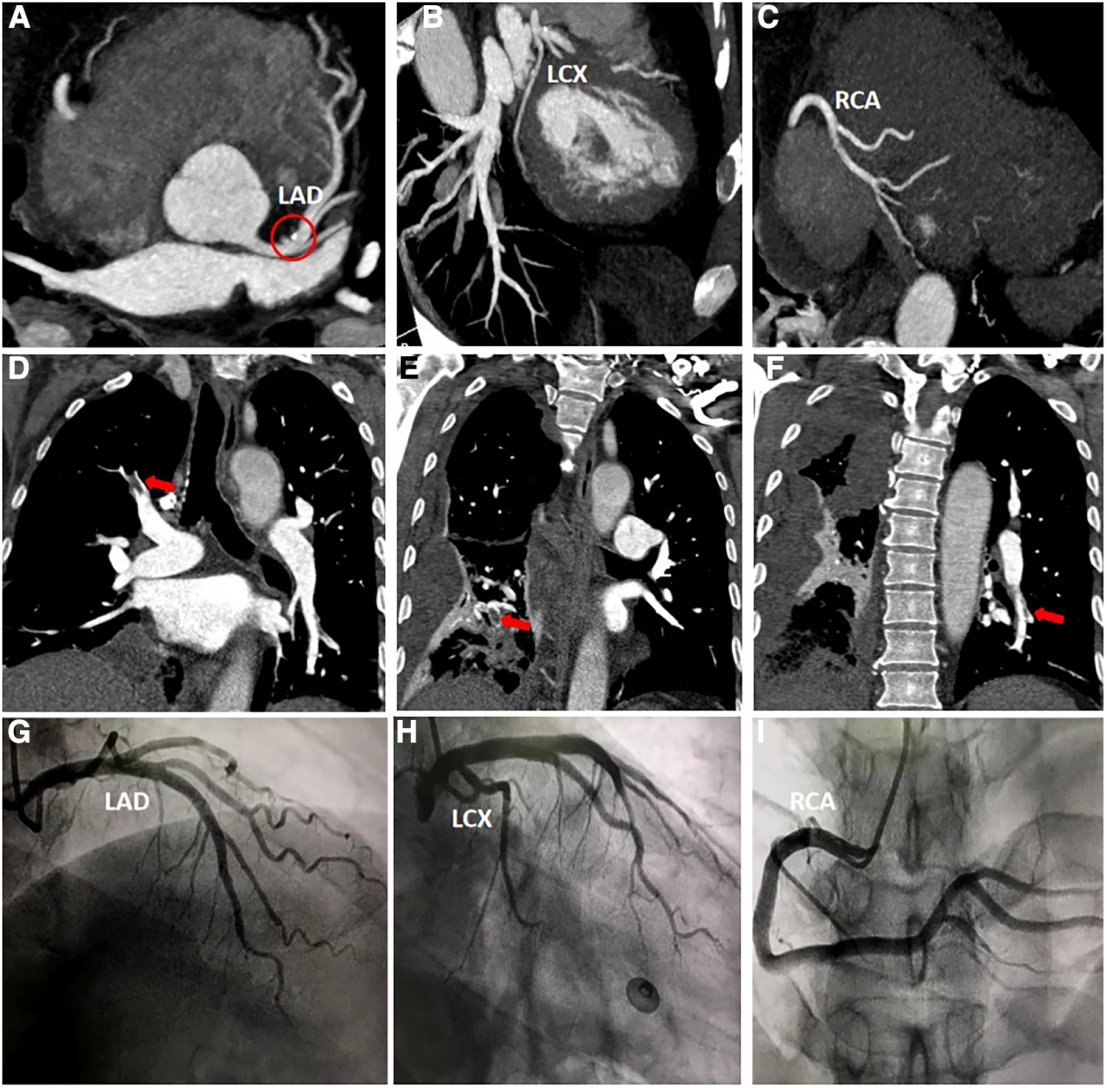
(**A–C**) Computed tomography coronary angiography scan. Calcification can be seen in the left anterior descending artery (red circle), and no other abnormalities were observed. (**D–F**) Computed tomography pulmonary angiography scan. The main findings revealed the presence of thrombi in the right upper lobe, right lower lobe, and left lower lobe. (**H–J**) Coronary angiography scan. The scan indicates that there are no significant narrowings or stenosis observed in either the left or right coronary arteries.

Furthermore, the patient was transferred to the Cardiology Department, where an emergency bedside echocardiography was performed. The echocardiogram showed that the diameters of the aorta and pulmonary arteries were within the normal range. Additionally, in the resting state, there were no definite segmental motion abnormalities observed in the left ventricular (LV) wall. Furthermore, there was no presence of pericardial effusion. The systolic function of the LV was found to be normal. At this point, ST segments of the inferior wall leads displayed resolution (Figure 1C), and the patient stated that the chest pain had subsided. Due to the presence of ICH, the Department of Neurosurgery suggested that anticoagulants should not be given for two weeks As a result, instead of performing a coronary angiography (CAG), we decided to proceed with a computed tomography coronary angiography (CTCA) to assess if there was any stenosis in the coronary artery. Surprisingly, the CTCA scan showed no signs of coronary artery occlusion or severe stenosis (Figures 3A–C). In addition, there were no identifiable factors present in the patient that could have caused coronary artery spasm. Furthermore, conditions such as AMI resulting from inadequate blood supply to the heart due to hypotension or anemia were ruled out.

However, a coagulation function test revealed a D-dimer level of 22.48 mg/L in the patient's blood. During the patient's hospitalization, the presence of hemoptysis and deep vein thrombosis (DVT) in their lower extremity were observed. Based on these symptoms, a possible diagnosis of PE was considered. To confirm this diagnosis, a computed tomography pulmonary angiography (CTPA) was performed (Figures 3D–F), and the diagnosis of PE was confirmed.

After successfully managing the ICH, the patient's condition stabilized, and he was discharged. He was prescribed oral warfarin for approximately 6 months, which gradually relieved his tightness in breathing symptoms. During his second hospitalization, a series of tests were conducted including biomarkers for myocardial injury, coagulation function assessment, echocardiography, CTPA and CAG. Importantly, the CTPA scan showed no abnormalities, indicating the absence of PE. Furthermore, the CAG results revealed no significant stenosis in the coronary arteries (Figures 3H–3J).

## Discussion

Patients who suffer from ICH often have restricted mobility and are frequently bedridden, which increases their vulnerability to developing venous thromboembolism (VTE). Notably, the incidence of VTE is particularly high among individuals with hemorrhagic stroke. It was found that the incidence of DVT in hemorrhagic stroke patients is nearly four times higher compared to those with thromboembolic stroke (2), with around 1.37% of patients with a hemorrhagic stroke had DVT, and 0.74% exhibited PE (1). Importantly, hemorrhagic stroke itself is recognized as an independent risk factor for the development of DVT (2). Moreover, there are additional factors that contribute to the risk of DVT, including advanced age, female gender, obesity, suspected infection, the presence of a central venous catheter, and notably, immobility resulting from paralysis or mechanical ventilation restrictions (3). In the present case, the patient had limited mobility in their right limb for a duration of six days, which could predispose them to the development of lower extremity DVT. DVT is one of the underlying causes of PE, which can be confirmed through a vascular ultrasound examination.

In fact, the presence of ST-segment elevation in leads II, III, and aVF, along with an increase in cTnT levels, initially posed a challenge in distinguishing PE from acute ST-segment elevation myocardial infarction (STEMI). This was further complicated by the occurrence of Ⅲ° AVB, a phenomenon that has not been previously reported in relevant literatures. Several factors may contribute to the observed ST-segment elevation. Firstly, PE-induced pulmonary artery hypertension (PAH) can cause acute right ventricular (RV) failure, resulting in impaired LV filling and subsequent hypotension (4). Secondly, the combination of hypotension, hypoxemia, PAH, and a surge in catecholamines may trigger transmural ischemia in RV. Additionally, a sudden increase in RV pressure may lead to myocardial cell stretching, resulting in ischemic injury (5). Thirdly, reduced coronary artery perfusion due to hypoperfusion resulting from increased RV pressure, can induce neurohormonal activation. The release of vasoconstrictors, such as endothelin, may further contribute to coronary spasm and cardiac ischemia. Therefore, it is hypothesized that PE can lead to a more severe increase in RV pressure, subsequently causing more severe ischemic injury, as reported in this case.

ECG plays a crucial role in both differentiating PE from other conditions and distinguishing it from AMI to some extent. In the case of PE, the presence of T-wave inversions and ST-segment depressions in leads V1–V3 is indicative, whereas these ECG changes in leads V4–V6 suggest AMI. However, in the current case, T-wave inversions and ST-segment depressions were observed in leads V1–V6, making the diagnosis more challenging. The occurrence of PE with Ⅲ° AVB is rare, with only a few reported cases found in the available literature (1, 6, 7). The occurrence of Ⅲ° AVB may be attributed to a preexisting left bundle branch block (LBBB). Notably, Ⅲ° AVB is the combination of a preexisting LBBB and an emerging RBBB. In the present case, the patient had a history of hypertension, which can cause ICH and LV hypertrophy, making him more likely to develop LBBB. However, it was difficult to determine whether the patient had RBBB or LBBB. Another possible explanation for the occurrence of Ⅲ° AVB could be ischemic injury to the atrioventricular node (AVN). The AVN receives its blood supply primarily from the AVN branch of the right coronary artery in approximately 90% of individuals (8). Therefore, the low perfusion of the coronary artery and potential coronary spasm could have led to dysfunction in the AVN, further contributing to the development of Ⅲ° AVB.

Large PE, particularly in the form of pulmonary artery trunk embolism or involving the left and right proximal pulmonary arteries, can at times present solely as III° AVB. In such cases, sudden death is often the main manifestation, making it challenging to save these patients if they do not reach the hospital in time. Consequently, encounters with cases of PE combined with III° AVB are relatively rare in clinical settings. In the case of the patient in question, the ECG obtained upon arrival at the emergency department demonstrated a sinus heart rate and ST elevation in the inferior wall. Additionally, the III° AVB had resolved, and the patient's dyspnea had improved. We speculated that the large embolus blocking the pulmonary artery may have turned into multiple small emboli because of bumpy transportation and that spontaneous fibrinolysis may be a possible etiology. This could explain the improvement of dyspnea and disappearance of Ⅲ° AVB.

In some cases, there is a risk of misdiagnosing AMI due to ECG findings that resemble AMI and elevated levels of cTnT. It is worth noting that the elevation of high-sensitivity cTnT (hs-cTnT) levels is the most specific and sensitive biomarker for diagnosing AMI. Therefore, it is recommended to dynamically monitor hs-cTnT levels in the management of acute coronary syndrome (9). However, it is important to recognize that any condition that causes myocardial injury can result in elevated hs-cTnT levels (10). Consequently, this factor alone cannot determine the exact cause of myocardial injury. For instance, patients with stroke and chronic kidney disease may have higher hs-cTnT levels compared to healthy individuals (11, 12). In the case of patients with PE, PAH, systemic hypotension, and hypoxia can lead to cardiac ischemia, resulting in elevated hs-cTnT levels. Therefore, it is crucial to carefully monitor and assess the dynamic changes in markers of myocardial injury.

Echocardiography also plays a crucial role in the rapid and accurate diagnosis of PE. As a quick, practical, and highly sensitive method for detecting RV overload following PE, echocardiography not only aids in early risk stratification but also serves as a valuable tool in differentiating PE from other potentially fatal conditions such as STEMI, pericardial tamponade, and aortic dissection (13). By using echocardiography, regional segmental ventricular wall dyskinesia can be identified. Specifically, in PE patients, it is possible to observe RV hypokinesia and/or dilation (14). In the case of our patient, during the emergency bedside echocardiography, attention was primarily focused on LV movements and functions, inadvertently neglecting the assessment of RV movements and functions. To ensure a comprehensive evaluation in the future, it is essential to include reporting on the RV movements and functions.

## Conclusions

Despite the complexity and challenges in diagnosing PE, the prognosis for this patient was positive. By utilizing the ECG and taking into account the patient's history of ICH and results of coagulation function, echocardiography, and CTPA, the misdiagnosis rate for PE can be reduced. It is essential to emphasize comprehensive clinical reasoning, multidisciplinary collaboration, and meticulous treatment and nursing care for patients with ICH in order to significantly decrease the diagnostic delay in cases like this.

## Data Availability

The raw data supporting the conclusions of this article will be made available by the authors, without undue reservation.
